# Applications of digital Medicine in oncology: Prospects and challenges

**DOI:** 10.1002/cai2.37

**Published:** 2022-12-04

**Authors:** Hewei Ge, Lixi Li, Di Zhang, Fei Ma

**Affiliations:** ^1^ Department of Medical Oncology, National Cancer Center/National Clinical Research Center for Cancer/Cancer Hospital Chinese Academy of Medical Sciences and Peking Union Medical College Beijing China

**Keywords:** digital medicine, oncology, artificial intelligence

## Abstract

The current state of oncology medical services is not encouraging and is unable to fully meet the needs of patients with cancer. In recent years, rapidly developing artificial intelligence technology and gradual advancements in mobile phones, sensors, and wearable devices, which have made these more compact, affordable, and popular, have greatly expanded the development of digital medicine. Digital medicine refers to clinical evidence‐based technology and products with a direct impact on disease management and research. Integrating digital medicine into clinical practice has the advantages of broader applicability, greater cost‐effectiveness, better accessibility, and improved diagnostic and therapeutic performance. Digital medicine has emerged in different clinical application scenarios, including cancer prevention, screening, diagnosis, and treatment, as well as clinical trials. Additionally, big data generated from digital medicine can be used to improve levels of clinical diagnosis and treatment. However, digital medicine also faces many challenges, including security regulation and privacy protection, product usability, data management, and optimization of algorithms. In summary, the application and development of digital medicine in the field of cancer face numerous opportunities and challenges.

## Graphical abstract

This review summarizes the applications of digital medicine in cancer prevention, screening, diagnosis, and treatment, as well as clinical trials. Integrating digital medicine into clinical practice has the advantages of broader applicability, greater cost‐effectiveness, better accessibility, and improved diagnostic and therapeutic performance.

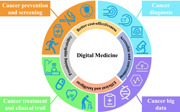

**Abbreviations**
AI: artificial intelligence
COVID–19: coronavirus disease 2019
DCT: decentralized clinical trial
DR: Deki Reader
IT: information technology
NLP: natural language processing
VR: virtual reality

## 1 BACKGROUND

An estimated 19.3 million new cancer cases and 10 million deaths from cancer were reported worldwide in 2020, with 28.4 million new cancer cases predicted in 2040, nearly a 50% increase over the number in 2020 [1]. With the increasing burden of cancer and aging populations worldwide, the shortage of medical resources has been magnified. Healthcare systems globally have long experienced uneven distribution of medical resources, a large gap between urban and rural medical care levels, and low coverage of cancer screening. In recent years, the rapid development of artificial intelligence (AI) and cloud computing technologies, as well as the optimization and popularization of mobile phones, sensors, and wearable devices [2], have accelerated the integration of digital technology into medical practice and the development of digital medicine in oncology [3].

The rise of digital medical care has provided an opportunity to address the shortage of medical resources, particularly during the pandemic of coronavirus disease 2019 (COVID‐19), which has reduced access to medical care. Digital medicine is highly convenient for doctors and patients and improves the efficiencies of diagnosis and treatment. However, uncertainties persist regarding how digital medicine can be applied to cancer screening, prevention, diagnosis, and treatment as well as the future trends. Here, we review the concept of digital medicine, advantages in clinical practice, and applications in different clinical oncology scenarios, summarize the research progress regarding digital medicine in oncology, and discuss its future development and challenges.

## 2 CONCEPT OF DIGITAL MEDICINE

Knowledge of digital medicine among many medical workers and patients is limited to remote consultation and online drug purchase. Currently, the definition of digital medicine is controversial, but definitions commonly include clinical evidence‐based technologies and products that will ultimately have a direct impact on the diagnosis, prevention, monitoring, and treatment of diseases, symptoms, and syndromes [2, 4, 5, 6]. Digital medicine is occasionally referred to as “mobile health” [7].

**Figure 1:**
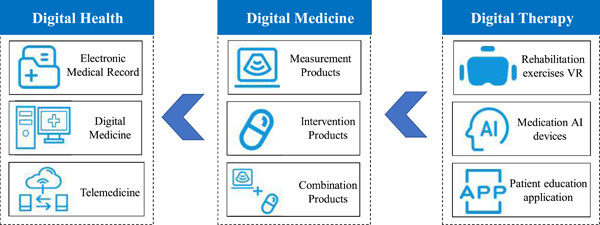
Link between digital health, digital medicine, and digital therapy. AI, artificial intelligence; VR, virtual reality.

Digital medicine is often confused with digital health and therapy; Figure 1 shows the relationship between them. Digital health includes digital medicine and can broadly be defined as anything that combines health care with digital technologies, including electronic medical record systems, telemedicine [8] used for Internet‐based diagnosis and treatment, and digital medicine [7, 9]. Digital therapy is a major part of digital medicine, including evidence‐based therapeutic interventions, such as virtual reality products to guide rehabilitation exercises and AI devices to guide medication [4].

Digital medicine products can broadly be classified into measurement, intervention, and combination products [2]. Measurement products include digital biomarkers (e.g., sound biomarkers tracking tremor changes in Parkinson's disease), electronic clinical outcome assessments (e.g., electronic patient‐reported outcome surveys), and tools used to measure adherence and safety (e.g., wearable sensors to track falls) [10]. Intervention products include digital therapeutics and connectable implantable devices (e.g., insulin pumps) driven by high‐quality programs providing patients with evidence‐based therapeutic interventions that can be used independently or in combination with drugs, devices, or other therapies [11]. Combination products combine assessment and intervention products. For example, the artificial pancreas combines a continuous glucose monitoring device with a computer‐regulated insulin pump, allowing the system to automatically adjust insulin infusion according to the patient's blood glucose level [12].

## 3 ADVANTAGES OF DIGITAL MEDICINE IN CLINICAL PRACTICE

**Figure 2:**
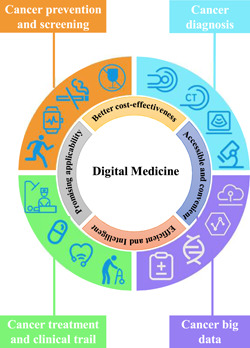
Applications and advantages of digital medicine in different fields of oncology

Integrating digital medicine into the traditional healthcare system has many advantages. Digital medicine has promising applicability, cost‐effectiveness, and accessibility and can improve the current level of medical care (Figure 2). Additionally, there are advantages to the data generated from digital medicine.

### 3.1 Promising applicability

Digital medicine has various potential applications and is flexible in practice, thereby complementing the current medical system well [13]. Digital medicine can be used alone or in conjunction with drugs, devices, or other products [2]. Moreover, it can be applied in both chronic disease management and emergency clinical situations [7]. For chronic disease management, digital medicine products can help educate patients and facilitate self‐management; in emergencies, digital medicine can help improve diagnosis and evaluation and facilitate communication between patients and healthcare workers.

### 3.2 Better cost‐effectiveness

Digital medical products are cost‐effective. Many systematic reviews have shown that, in most cases, digital medicine is cost‐effective for the diagnosis and treatment of diabetes [14], mental illness [15], and cardiovascular disease [16]. Approximately 50% of costs can be saved without reducing benefits [15, 16]. Although some digital medicine products will increase costs, these can save patients a substantial amount of the time spent on accessing medical care. For example, a systematic review in skin cancer showed that when using teledermoscopy for skin cancer referral and triage, each case cost an additional USD 54.64 on average, but medical services were received 26 days sooner than usual [17].

### 3.3 Better accessibility

Digital medicine can improve people's accessibility to medical resources, facilitate patient visits, and save time. In addition to teledermoscopy, digital medicine can save on the time spent in visiting a clinic and is convenient for patients working in remote areas [18, 19] because medical care is not limited by spatial location. This also helps to more evenly distribute medical resources and promote medical equity.

### 3.4 Improved medical care quality

Digital medicine products can improve the quality of medical care in many ways and help promote the homogeneous development of medical services in various regions. It can aid physician decision‐making. In a study that investigated the use of a mobile device—the Deki Reader (DR)—to aid processing and interpreting of rapid diagnostic tests for malaria, the results showed a consistency rate of 91.6% between the DR and community health workers, with decreased error probability with increased number of tests [20]. Digital medicine can also be used to perform many screening tests for various diseases, thereby promoting personalized screening and overcoming drawbacks of the current medical paradigm in which patients often visit a doctor only after symptoms appear. For example, digital medicine can make use of mobile devices with physiological sensors to continuously monitor peoples' heart rate, blood pressure, body temperature, blood sugar, and other indicators for earlier diagnosis and treatment of diseases, such as hypertension and diabetes [5]. Additionally, digital medicine can be used as a treatment method, also called digital therapy, to manage patients remotely and improve patient outcomes, such as via cognitive behavioral therapy or using digital products to alleviate depression and anxiety in perinatal women, as in one study [13].

### 3.5 Valuable big data

Data generated in digital medicine have numerous advantages. First, digital medicine devices can generate abundant real‐world data that are unavailable via existing medical paradigms, also called “digital phenotyping”, such as data from continuous monitoring of patients through remote sensors. These data can be analyzed alone or in combination with other data to build more comprehensive disease models [5]. A study that examined continuous monitoring data in patients with Alzheimer's disease, including lifestyle and cognitive ability, revealed the relationship with baseline amyloid levels [21]. Additionally, abundant data generated by digital medicine can be used for quality control in disease diagnosis and treatment.

In conclusion, digital medicine can be used as a platform for evidence implementation, to perform various existing medical interventions, [19] and data generation for scientific research and quality control. Digital medicine is increasingly being used for various diseases. We focus on its application in oncology in detail.

## 4 CURRENT APPLICATIONS OF DIGITAL MEDICINE IN ONCOLOGY

Digital medicine can be applied in various clinical scenarios, such as tumor prevention, screening, diagnosis, treatment, management, and follow‐up (Figure 2; Table 1). The generated data can also be used for scientific research, clinical quality control, and for other purposes, which is helpful for resolving current tumor‐related medical problems. Cross‐scale and multimodal biomedical big data suggest the benefits of common key technologies in fusing biomedical information and establishing a digital AI diagnosis and treatment platform covering all aspects and full cycles of tumors.

**Table 1:** Applications, effects, and examples of digital medicine

Application fields	Effects	Examples
Tumor prevention	Promoting healthy behavior	Weight loss software
		Ultraviolet monitoring device
Tumor screening	Increasing screening awareness	Education, awareness, and reminder software
	Personalized screening	Web‐based risk‐assessment chatbot
	More biomarkers	Digital biomarkers detected by sensors or inferred by algorithms
Tumor diagnosis	Remote diagnosis	Mobile teledermatology
	Auxiliary diagnosis algorithms	Skin disease diagnosis mobile application
Tumor treatment	Remote management	Breast cancer self‐management game
		Web‐based self‐management mobile application
Clinical trial management	Precise patient recruitment	IT platform using AI and NLP to select patients
	Promoting patient management	Patient‐centered and real‐time data recording DCT
Tumor big data	New digital phenotype	Features derived from continuous monitoring data of patients
	Scientific research	Finding more evidence
	Clinical quality control	Quality control platforms to help make public decisions

Abbreviations: AI, artificial intelligence; DCT, decentralized clinical trial; IT, information technology; NLP, natural language processing.

### 4.1 Cancer prevention

Numerous digital medical products have been developed and put into practice to promote healthy behaviors in the population, including healthy eating, exercising, sun protection, avoiding harmful substances (mainly alcohol and tobacco), and addressing mental illness [19, 22]. Several countries have treated digital medicine as a potentially efficient solution to the burden of cancer and general noncommunicable disease prevention and control [23]. A typical application of digital medicine in tumor prevention is weight loss. A meta‐analysis exploring the effect of mobile phone applications on weight loss found that using an application was significantly associated with weight loss and reduction of body mass index, demonstrating the feasibility of using mobile phone applications to guide weight loss and thereby prevent obesity‐related tumors [24]. Another typical application is sun protection. A study on the correlation between wearing an ultraviolet (UV) monitoring device and UV exposure showed that outdoor activity time and sunscreen use time among parents and children changed significantly after wearing the monitoring device for 2 weeks [25], highlighting the potential effect of preventing skin cancer using wearable UV monitoring devices.

### 4.2 Cancer screening

Digital medicine can facilitate tumor screening in several ways. First, digital medical products can increase screening awareness and coverage among the general public. A 2021 study that evaluated the effectiveness of digital medicine products in facilitating screening included multiple types of digital medicine interventions, such as peer support (*n* = 1), education and raising awareness (*n* = 6), reminders (*n* = 13), and mixed interventions (*n* = 19). The results showed a combined odds ratio of 1.49 (95% confidence interval: 1.31–1.70), which was similar across cancer types [26]. Therefore, using different forms of digital medicine, health awareness among the public can be improved and participation in cancer screening can be promoted. Furthermore, use of digital medicine can facilitate early diagnosis and treatment of cancer.

Second, digital medicine can facilitate personalized screening of tumors. With the rapid development of big data technology and machine learning/AI, better understanding of tumors and advancements in digital medicine products, personalized screening algorithms have increasingly been put into clinical practice [19]. For example, a 2021 study on a chatbot showed that women's cancer risk could be predicted in advance [27]. One‐quarter of participants screened using the chatbot met the USA National Comprehensive Cancer Network genetic testing criteria, and 5.6% showed a pathogenic variant in subsequent genetic testing. This suggested that the chatbot could intelligently identify people at a high risk for hereditary cancer syndromes and formulate personalized and preventive genetic testing plans.

Digital medicine products can generate digital biomarkers for early‐stage cancer screening. Similar to the continuous monitoring of heart rate, blood pressure, body temperature, and blood sugar can be monitored using physiological sensors [5]. In the future, a noninvasive sensor can monitor specific tumor targets, such as tumor markers in peripheral blood, circulating tumor cells, and cell‐free DNA.

### 4.3 Cancer diagnosis

Digital medical products can be used for remote tumor diagnosis, which is also called Internet medicine. In an oral cancer study in India, front‐line healthcare workers examined patients and uploaded images to the Internet for remote experts to provide an initial diagnosis and screen individuals with a higher risk of malignancy. The accuracy rate was 0.8%–62% and the cost was less than USD 1 per person [28]. That study used Internet‐based medical care to conduct tumor risk assessment and diagnosis for remote populations in a cost‐effective way, showing the potential applications of Internet‐based medical care in remote areas. In a skin cancer study in Switzerland, the efficacy of mobile teledermatology (with or without dermoscopy images) as a screening test was assessed using histopathology as the gold standard. The study findings showed 100% sensitivity and 77% specificity without dermoscopy, which increased to 85% with dermoscopy, highlighting the good diagnostic performance of Internet medicine [29].

Digital medicine algorithms can assist physicians in diagnosing diseases. In a dermatological study in India, a digital medicine product to diagnose various common skin diseases, including skin cancer, was developed and clinically validated. The results showed an accuracy rate of 89.62% for patients with pigmented skin [30]. Therefore, digital medicine is promising as an efficient and accurate clinical decision support tool to assist clinicians in tumor diagnosis, thereby saving workforce and time costs.

### 4.4 Cancer treatment

Using digital medicine products for treatment is called digital therapy, and the current application in clinical oncology mainly pertains to managing patients remotely. A smartphone game that helps breast cancer patients with chemotherapy self‐management has been launched internationally. It has shown good results in a clinical trial: the game group showed better medication compliance than the traditional education group, showing a lower incidence of chemotherapy‐related side effects and improved quality of life [31]. A 2019 systematic review also reported many encouraging results regarding the application of digital medicine in breast cancer management [32]. However, not all digital therapy products benefit patients. A 2020 study on Oncokomps, a web‐based digital application that assists patients with self‐management, showed no significant differences in self‐management knowledge, skills, or confidence between the intervention and control groups. Many cases of chronic disease have been managed successfully with digital therapy, including glycemic control of diabetes and self‐management of patients with mental illness. However, applications in oncology require more medical evidence [33].

### 4.5 Clinical trial management

Digital medicine technologies can help in managing clinical trials. The information technology platform has been used to assist precise patient recruitment. The platform can use AI and natural language processing (NLP) to identify the enrollment criteria, institutional patient population, and personal electronic medical records as well as assist in selecting the clinical trial site and in the recruiting and screening of patients [34]. Another typical case is that of decentralized clinical trials (DCT). DCT have many advantages, such as a patient‐centered approach, enhanced participant recruitment, and real‐time data recording [35]. However, DCT also face some challenges. A review published in 2022 pointed out the challenges in oncology research. When patients encounter a disease emergency during DCT, they need intervention from local doctors; however, because they are not involved in the trial, local doctors cannot intervene in accordance with the study criteria. Additionally, whether DCT allow patients to be treated at home and how to effectively ensure patients' safety are also urgent issues to be solved [36]. Further exploration and improvement of DCT products are required.

### 4.6 Tumor big data

Digital medicine products can generate massive amounts of data, thereby generating great potential value. A 2021 review showed that the data generated from digital medicine can be used for disease diagnosis and prediction [37]. The concept of “digital phenotype” argues that the analysis of longitudinal data through continuous monitoring may reveal new markers that provide information about disease severity or progression and may create new opportunities for oncology research [5]. Moreover, these big data may be used for quality control in tumor diagnosis and treatment. Analyzing differences in the medical care level across regions can help policy makers in planning health services.

## 5 CHALLENGES

Currently, digital medicine faces numerous challenges, one of which pertains to security supervision and privacy protection. Although digital medicine has been greatly advanced in the field of oncology over the past few years [38], many digital products do not incorporate evidence‐based functions [19], and strong regulations and legislations for data security, privacy, and digital ethics are currently lacking.

The second challenge is the usability of digital medicine products. These products should be patient‐centered. Many vulnerable populations, such as elderly people and those in remote areas, have limited access to resources and cannot easily adapt to complex digital medicine systems. Therefore, digital medicine products should be designed that can be generalized to more populations, which can also promote social equity [39]. Additionally, most complex digital medicine products for chronic or acute diseases are not cost‐effective, underscoring the importance of simplicity and ease of use [7]. Additionally, interactive formats should be considered, such as games, dialog, and classes, which may be applicable to different groups and diseases.

The third challenge is data management. On the one hand, reasonable data standards should be formulated to facilitate subsequent processing, sharing, and analysis of the generated data. On the other hand, integrating patient data from different systems and comprehensively analyzing patients' conditions for decision‐making remain challenging. These may be achieved by setting an orderly and secure workflow for data exchange between emerging digital medicine applications and traditional electronic medical record systems, such that the two systems can complement each other [17].

Finally, the optimization of algorithms has certain difficulties, and most research findings have not been translated into actual practice in clinical diagnosis and treatment. Many digital medicine products have not shown significant benefits [33]. Machine learning has undergone considerable progress in tumor detection [19, 40], deep learning is superior to humans in reading digital X‐ray images [41], and AI has gradually transformed genomics data into precision medicine applications [42]. However, many challenges remain in providing personalized digital services to patients, obtaining accurate patient diagnoses, and proposing reasonable intervention recommendations. This is partly owing to an insufficient understanding of tumors, although a main reason is that the current algorithms are not well adapted to the massive dimensions of digital medicine data generated in the real world, resulting in unsatisfactory performance of AI, also called “the curse of dimensionality” [43]. Therefore, new AI algorithms should be developed to adapt to real‐world data and improve prediction performance.

## 6 CONCLUSIONS

Digital medicine has been applied to the full‐cycle management of cancer, including cancer prevention, screening, diagnosis, treatment, and scientific research, as well as other fields. Digital medicine has certain advantages in terms of cost‐effectiveness, medical resource accessibility, and medical care improvement. However, challenges include insufficient security supervision and privacy protection, low data management efficiency, unsatisfactory product usability, and low‐performance algorithms. Therefore, digital medicine should be improved and innovations developed in consideration of the oncology conditions in different countries and regions. Customized digital medicine products should be developed, as well as integrated digital AI diagnosis and treatment platforms that cover the entire tumor treatment cycle.

## AUTHOR CONTRIBUTIONS

**Hewei Ge**: Data curation (equal); investigation (equal); methodology (equal); visualization (equal); writing – original draft (equal); writing – review and editing (equal). **Lixi Li**: Data curation (equal); investigation (equal); methodology (equal); visualization (equal); writing – original draft (equal); writing – review & editing (equal). **Di Zhang**: Data curation (supporting); investigation (supporting); methodology (supporting); visualization (supporting); writing – original draft (supporting); writing – review and editing (supporting). **Fei Ma**: Conceptualization (lead); funding acquisition (lead); writing – review and editing (lead).

## CONFLICT OF INTEREST

Professor Fei Ma is a member of the *Cancer Innovation* Editorial Board. To minimize bias, he was excluded from all editorial decision‐making related to the acceptance of this article for publication. The authors declare no conflict of interest.

## ETHICS STATEMENT

Not applicable.

## INFORMED CONSENT

Not applicable.

## Data Availability

Data sharing is not applicable to this article as no new data were created or analyzed in this study.
